# In Vitro Antioxidant Activities of Methanolic Extracts of *Caesalpinia volkensii* Harms., *Vernonia lasiopus* O. Hoffm., and *Acacia hockii* De Wild

**DOI:** 10.1155/2020/3586268

**Published:** 2020-09-27

**Authors:** Beatrice Muthoni Guchu, Alex King'ori Machocho, Stephen Kiruthi Mwihia, Mathew Piero Ngugi

**Affiliations:** ^1^Department of Biochemistry, Microbiology and Biotechnology, School of Pure and Applied Sciences, Kenyatta University, P. O. Box 43844-00100, Nairobi, Kenya; ^2^Department of Chemistry, School of Pure and Applied Sciences, Kenyatta University, P. O. Box 43844-00100, Nairobi, Kenya

## Abstract

Oxidative stress is the result of the disparity between pro-oxidants and antioxidants in an organism, and it is important in the pathogenesis of several degenerative disorders, such as arthritis, Alzheimer's, cancer, and cardiovascular diseases. Free radicals can damage biomolecules, such as nucleic acids, lipids, proteins, polyunsaturated fatty acids, and carbohydrates, and the DNA leading to mutations. The use of antioxidants is effective in delaying the oxidation of biomolecules. Antioxidants are complexes found in the food that can retard or deter oxidation by preventing the initiation and propagation of oxidizing chain reactions. Medicinal plants have been used for centuries by man to manage diseases and have a host of antioxidant complexes. Traditionally, *Caesalpinia volkensii, Vernonia lasiopus,* and *Acacia hockii* have folkloric remedies against associated oxidative stress-mediated complications. However, the upsurge in its use has not been accompanied by scientific validations to support these claims. In this study, in vitro antioxidant activity of *Caesalpinia volkensii, Vernonia lasiopus,* and *Acacia hockii* collected from Embu County (Kenya) were determined by radical scavenging activities of 1,1-diphenyl-2-picrylhydrazyl (DPPH) and hydroxyl radical in addition to ferric reducing antioxidant power analyzed against that of L-ascorbic acid as the standard. The obtained results revealed remarkable antioxidant activities of the studied plant extracts as evidenced by the low IC50 and EC50 values. These antioxidant activities could be due to the presence of antioxidants phytochemicals such as flavonoids, phenols, terpenoids, and saponins among others. Therefore, the therapeutic potential of this plant could be due to their antioxidant properties. This study recommends bioassay of the extracts against oxidative stress-related disorders for development of phytomedicine with antioxidant properties.

## 1. Introduction

Oxidative stress is the major driving factor responsible for the initiation and progression of cancer, diabetes mellitus, cardiovascular diseases, neurodegenerative diseases, and inflammatory diseases among other syndromes [1]. The condition is brought by excessive generation of free oxygen and nitrogen species or their inefficient quenching in the cell. Free oxygen and nitrogen species are unstable molecules that are present in the environment (exogenous) and are also generated in the body (endogenous) during the normal aerobic metabolic processes in the body [2]. Exogenous sources of free radicals include cigarette smoke, exposure to ozone, ionizing radiation such as X-rays, and drugs among others. On the other hand, endogenous sources of free radicals include the electron transfer chain reactions in the mitochondria, xanthine oxidase pathway, during disease states such as inflammation, ischemia, and reperfusion injury [3].

The body possesses a complex antioxidant defense system, comprising of enzymatic and nonenzymatic pathways, which in the normal physiologic state, maintain a steady equilibrium between prooxidants and antioxidants, thereby ensuring well-being [1]. The enzymatic antioxidants comprise the catalase, glutathione peroxidase, and superoxide dismutase. Conversely, nonenzymatic antioxidants employed by the body include the bilirubin, uric acid, and lactoferrin among others. However, during disease states, the endogenous antioxidant systems are overwhelmed, leading to accumulation of excessive free radicals, which in turn cause oxidative stress-associated damage to cellular machinery, as implicated in various diseases [4].

Conventionally, oxidative stress is managed using various synthetic antioxidant compounds such as butylated hydroxyanisole (BHA), butylated hydroxytoluene (BHT), and propyl gallate (PG). Despite their usage, these synthetic antioxidant compounds have been associated with undesirable effects [5]. For instance, BHT and BHA cause hepatotoxicity and have been demonstrated to be carcinogenic. Additionally, synthetic antioxidants are inaccessible, unaffordable, and labile, thus limiting their utilization [3]. Therefore, due to the profound consequences of oxidative stress and the drawbacks of synthetic antioxidants, the need for alternative antioxidants, which are safer, easily accessible, and potent, are warranted [6], hence the current study.

Considering the available alternative and complementary strategies, medicinal plants stand a better chance of providing potent, safer, affordable, and easily accessible therapies for oxidative stress-related maladies [7]. Medicinal plants contain various secondary metabolites, which have demonstrated a wide spectrum of pharmacologic activities. Antioxidants properties of plants have been demonstrated to play a protective role in the body against diseases, since their consumption lowers the risk of cancer, heart disease, hypertension, dementia, and stroke [8].

The major groups of phytochemicals that contribute to antioxidant capacity of plants include polyphenols and vitamins (A, C, and E). Phenolic compounds of plants are hydroxylated derivatives of benzoic acid and cinnamic acids, which possess antioxidant and anticarcinogenic effects [3]. They include phenols, flavonoids, coumarins, tannins, and anthocyanidins. These phytoactive complexes are important in plant defense mechanisms against biotic and abiotic stresses [9]. When plants or plant products rich in these phytoactive principles are consumed, they are deemed to confer the same beneficial effects to humans [8]. For instance, flavonoids have for long been recognized to possess anti-inflammatory, antiallergic, antiviral, immunomodulatory, antiaging, and antiproliferative properties [10].

The search for better alternatives to synthetic antioxidants has triggered a significant research interest on dietary and medicinal plants that can inhibit, reverse, or ameliorate diseases caused by oxidative stress [3, 10]. In this quest, we investigated the in vitro antioxidant activities of the methanolic extracts of *Vernonia lasiopus* (O. Hoffm.), *Acacia hockii* (De Wild.), and *Caesalpinia volkensii* (Harms.). *Vernonia lasiopus* (O. Hoffm.) is a shrub of Asteraceae family. Leaf infusion and decoctions of *V. lasiopus* are used by traditional herbalists in Kenya for the treatment and management of malaria, epilepsy, inflammation, pain, and other diseases [11, 12]. Previous studies have implicated various plants of the Asteraceae family to have antioxidant activities. For instance, Iwalokun et al. [13] reported the antioxidant effects of an aqueous extract of *Vernonia amygdalina* leaves against acetaminophen-induced hepatotoxicity and oxidative stress in mice.

*Acacia hockii* (De Wild.) is a shrub in the Fabaceae family. The root and back decoctions of *A. hockii* are used in traditional medicine to treat inflammation and associated diseases including gout [12]. Other plants of genus *Acacia,* in the Fabaceae family, have been shown to have harbor antioxidant properties. An investigation of one of the *Acacia* species, *Acacia mearnsii*, has led to the isolation of a strong antioxidant molecule, with neuroprotective properties [14].

*Caesalpinia volkensii* (Harms.) is a shrub belonging to the Caesalpiniaceae family. Fruit and leaf preparations of this plant are used by herbalists to manage diarrhea, dysentery, ophthalmic diseases, and diabetes mellitus complications [12]. Various plants in Caesalpiniaceae family, including *Bauhinia rufescens,* have antioxidant properties as described by Aliyu et al. [15]. In this study, *Caesalpinia volkensii, Acacia hockii,* and *Vernonia lasiopus* were selected based on their ethnomedical usage in the management of oxidative stress-related diseases. Thus, the current study provides a framework towards the discovery of alternative, safer, affordable, and efficacious antioxidants to counter oxidative stress-related disorders.

## 2. Materials and Methods

### 2.1. Plant Materials and Processing

Fresh leaves of *Caesalpinia volkensii* and *Vernonia lasiopus* and stem bark of *Acacia hockii* were collected from their natural habitats, in Mbeere North subcounty, Embu County, where they grew naturally. The plants were chosen based on an extensive ethnomedical survey and folklore reports from the local practicing herbalists [12]. Primarily, the plants were identified by their local names, *Caesalpinia volkensii* (“Mubuthi”), *Vernonia lasiopus* (“Mucatha”), and *Acacia hockii* (“Mugaa”), and diseases they treat by the help of a reputable local herbalist.

The plant samples were furnished to an acknowledged taxonomist for botanical authentication. The voucher specimens were deposited in plant science department of Kenyatta University for future reference. Samples were sorted out properly and transported to the Department of Biochemistry, Microbiology and Biotechnology laboratories at Kenyatta University where they were shade dried for a period of two weeks. After drying, the plant materials were ground well using an electric plant mill into a fine powder, packaged in well-labeled airtight containers, and stored at room temperature awaiting extraction.

### 2.2. Preparation of Methanolic Extracts

Approximately 400 g of each of the powdered plant materials was soaked in a liter of analytical grade methanol in a 2-liter capacity conical flask. The flasks containing each plant material were shaken regularly, corked, and left to stand for 48 hours at room temperature. In each case, the menstruum was separated by filtration through Whatman filter paper No. 1. The filtrates were then concentrated using a rotary evaporator at 50°C and later in a hot-air oven at 35°C to dry completely. The concentrates were put in airtight containers and stored at 4°C awaiting use in in vitro bioassay [16].

### 2.3. Qualitative Phytochemical Screening

Qualitative tests for various phytochemicals present in the methanolic leaf extracts of *Caesalpinia volkensii* and *Vernonia lasiopus* and the methanolic stem bark extract of *Acacia hockii* were carried out using standard phytochemical screening procedures [16]. Visual examination of the appearance of colour or frothing was used as an indicator for the presence or absence of a given phytochemical group.

#### 2.3.1. Test for Saponins

About 2 g of each of the studied plant extracts was weighed and dissolved in 5 ml of distilled water. Thereafter, aliquots of 2 ml were taken from each plant extract solution, stirred for 30 seconds, and briskly agitated. The setups were then allowed to settle for 15 minutes. The presence of frothing, which persists for over 15 minutes, is an indication of the presence of saponins in the tested sample [16].

#### 2.3.2. Test for Alkaloids

About 2 g of each of the studied plant extracts was added to 10 ml of 0.1 M hydrochloric acid, warmed in a waterbath (50°C) for 5 minutes, and filtered through Whatman filter paper No. 1. After cooling, 3 drops of Dragendorff's reagent were added and mixed. The appearance of a reddish-brown colour is a positive indication for the presence of alkaloids in the sample [16].

#### 2.3.3. Test for Terpenoids

Into clean test tubes, 2 ml of alcoholic extracts were mixed with 5 drops of acetic anhydride. Thereafter, 5 drops of concentrated sulphuric were carefully added through the side of the test tube.

The formation of a blue ring at the interface shows the presence of terpenoids in the tested sample [16].

#### 2.3.4. Test for Flavonoids

To 2 ml of alcoholic extracts of the studied plants and 5 drops of concentrated hydrochloric acid were added. The formation of a red colour indicates the presence of flavonoids. To another portion of the alcoholic extracts (2 ml), 1 ml of dilute ammonia was added and gently mixed. A greenish-yellow colour indicates the presence of flavonoids [16].

#### 2.3.5. Test for Cardiac Glycosides

To test for cardiac glycosides presence, 0.5 g of the extract was dissolved in 2 ml glacial acetic acid containing 2 drops of 10% ferric chloride solution. One milliliter of concentrated H_2_SO_4_ was then slowly introduced into the underlying mixture. Appearance of either a violet band at the boundary is a positive test for the deoxy sugars (cardenolides) [16].

#### 2.3.6. Test for Steroids

The presence of steroids in the studied plant extracts was determined in this study. About 0.5 g of each extract was dissolved in 2 ml of chloroform. This was followed by addition of 3 drops of the Liebermann–Burchard reagent and gently agitated. The presence of reddish-purple colour indicates the presence of steroids [16].

#### 2.3.7. Test for Phenols

About 0.5 g of each of the studied plant extracts was boiled in 5 ml of 70% ethanol in a waterbath for 5 minutes and then filtered through Whatman filter paper No. 1. After cooling, 5 drops of 5% ferric chloride were added and mixed. The appearance of a green precipitate indicates the presence of phenols in the sample [16].

### 2.4. Determination of In Vitro Antioxidant Activities of the Studied Plant Extracts

#### 2.4.1. Ferric Reducing Antioxidant Power Assay

The reducing power of the extracts was determined according to the method described by Oyaizu [17], with some modifications. Briefly, five different concentrations of methanolic extracts (0.2, 0.4, 0.6, 0.8, and 1 mg/ml) and L-ascorbic acid at same concentrations were mixed with 2 ml phosphate buffer (0.2 M, pH 6.6) and 2 ml of 1% potassium ferricyanide (K_3_Fe (CN)_6_). The mixture was incubated at 50°C for 20 minutes. Then, 2 ml of 10% trichloroacetic acid (TCA) was added, and the mixture was centrifuged at 1000 revolutions per minute (rpm) for 10 min. The supernatant (2 ml) was aspirated and mixed with 2 ml of distilled water and 1 ml of 0.1% ferric chloride (FeCl_3_). In each case, the experiment was performed in triplicate. Afterward, the absorbances were measured spectrophotometrically at 700 nm using a UV-vis spectrophotometer and recorded. The concentrations of each extract able to yield an absorbance value of 0.5 were determined from the graph of absorbance at 700 nm against extract concentrations and considered as the median effective concentration (EC50).

#### 2.4.2. Determination of 1,1, dipheny-2-picrylhydrazyl (DPPH) Radical Scavenging Activities

The DPPH radical scavenging assay was performed using 1,1 diphenyl-2-picrylhydrazyl (DPPH) according to the method described by Brand-Williams et al. [18] with some modifications. Briefly, five different concentrations of the studied plant extracts (0.0625, 0.125, 0.25, 0.5, and 1 mg/ml) were prepared in methanol (analytical grade). The same concentrations were also prepared for L-ascorbic acid, which was used as a standard antioxidant. 1 ml of each studied extract was transferred into a clean test tube into which 0.5 ml of 0.3 mM DPPH in methanol was added. The mixture was shaken and left to stand in the dark at room temperature for 15 minutes. Blank solutions comprising of the studied extract solutions (2.5 ml) and 1 ml of methanol were used as baseline.

The negative control comprised 2.5 ml of DPPH solution and 1 ml of methanol, while L-ascorbic acid at the same concentrations as the studied extracts was used as the positive control. After incubation in the dark, the absorbance values were measured at 517 nm using a spectrophotometer. The experiments were performed in triplicate. The DPPH radical scavenging activity was estimated using the equation described by Brand-Williams et al. [18].

(1) $$\begin{array}{c} \%\text{ Radical scavenging activity}=\frac{A_{\text{c}}-A_{\text{s}}}{A_{\text{c}}}\times 100, \end{array}$$

where *A*_s_ is the absorbance of the sample, and *A*_c_ is the absorbance of the control.

The half maximal inhibitory concentration (IC50) of the extracts was computed from a plot of percentage DPPH free radical inhibition versus the extract concentration.

#### 2.4.3. Hydroxyl Radical Scavenging Activities

The hydroxyl radical scavenging activity was performed as per the method described by Klein et al. [19] with minor modifications. The reaction mixture was constituted by adding 2.4 ml of phosphate buffer (pH 7.8) into test tubes. To the same test tubes, 90 *μ*l of 1 mM 1, 10 phenanthroline, 150 *μ*l of 0.1 mM hydrogen peroxide, 60 *μ*l of 1 mM iron (III) chloride, and 1.5 ml of the Phytexponent and the standard (L-ascorbic acid) at different concentrations (100%, 10%, 1%, 0.1%, and 0.01%) were added except in the controls, followed by incubation at room temperature for 5 minutes. The increase in absorbance at 560 nm was measured, and radical scavenging activity was calculated using the following formula:

(2) $$\begin{array}{c} \%\text{ Radical scavenging activity}=\left[\frac{\text{Abs of control}-\text{Abs of sample}}{\text{Abs of control}}\right]\times 100. \end{array}$$

### 2.5. Determination of Total Phenolic Contents

The total phenolic content of the extracts was measured according to the Folin-Ciocalteu method adapted from Do et al. [20], with some modifications. Briefly, the extract (1 ml) was mixed with 2 ml of Folin-Ciocalteu reagent, which was prepared by dilution with distilled water in a ratio of 1 : 10 v/v, after which 1 ml of 20% sodium carbonate (Na_2_CO_3_) was added. The mixture was shaken for 20 seconds and incubated at 40°C for 30 minutes. Absorbance was measured at 765 nm. Gallic acid was used for the generation of the standard curve. The total phenolic content was expressed as mg of gallic acid equivalents (GAE) per gram (g) of the studied extracts. The experiment was repeated in triplicates.

### 2.6. Determination of Total Flavonoid Contents

The total flavonoid content of the extracts was evaluated through a technique described by Park et al. [21]. In a 10 ml test tube, 0.3 ml of extracts, 3.4 ml of 30% methanol, 0.15 ml of NaNO_2_ (0.5 M), and 0.15 ml of AlCl_3_·6H_2_O (0.3 M) were mixed. After 5 minutes, 1 ml of NaOH (1 M) was added and mixed well, and the absorbance was measured against the reagent blank at 510 nm. The standard curve for total flavonoids prepared using quercetin standard solution (0–100 mg/l). The total flavonoids were expressed as milligrams of quercetin equivalents per g of sample. The experiment was repeated thrice.

### 2.7. Data Management and Statistical Analysis

Quantitative data were presented in tables, and the data were then exported into Minitab statistical software version 17.0 for analysis. The data were subjected to descriptive statistics and stated as mean ± standard error of the mean (SEM). One-way analysis of variance (ANOVA) was used to analyze whether there was any significant difference among the means of different groups. This was followed by Tukey's tests for pairwise comparisons and separation of means. *P* < 0.05 was considered statistically significant. On the other hand, qualitative data on phytochemical analysis was only tabulated.

## 3. Results

### 3.1. Qualitative Phytochemical Screening

Qualitative phytochemical testing of the studied plant extracts showed the presence of saponins, terpenoids, flavonoids, alkaloids, and phenols. However, *C. volkensii* and *V. lasiopus* lacked cardiac glycosides and steroids (Table 1).

### 3.2. Antioxidant Activity

#### 3.2.1. Ferric Reducing Antioxidant Power (FRAP)

Generally, the methanolic extracts of three studied medicinal plants exhibited remarkable concentration-dependent increases in absorbance values at a wavelength of 700 nm (Table 2). At all the tested concentrations, the extracts of *A. hockii* had significantly higher absorbance values than those recorded for *C. volkensii* and *V. lasiopus* extracts (*P* < 0.05; Table 2). Notably, at all the tested concentrations, the methanolic extract of *V. lasiopus* produced significantly higher absorbance values compared with the absorbance generated by *C. volkensii* extracts (*P* < 0.05; Table 2). However, the standard (L-ascorbic acid) demonstrated significantly higher absorbances than those obtained for the three studied plant extracts (*P* < 0.05; Table 2). Furthermore, the half effective concentrations (EC50) of the studied plant extracts required to produce an absorbance value of 0.5 were determined in this study. It was demonstrated that the EC50 value for L-ascorbic acid was lower than the EC50 values of all the studied plant extracts. However, the methanolic extract of *A. hockii* had the lowest EC50 value compared with the EC50 values of *C. volkensii* and *V. lasiopus* (Table 2). Furthermore, it was observed that methanolic extracts of *V. lasiopus* had lower EC50 values than *C. Volkensii* (Table 2).

#### 3.2.2. DPPH Radical Scavenging Activities of Methanolic Extracts of *C. volkensii, V. lasiopus,* and *A. hockii*

The methanolic extracts of *C. volkensii, V. lasiopus,* and *A. hockii* also demonstrated remarkable in vitro DPPH radical scavenging activities in a dose-dependent trend (Table 3). The standard (L-ascorbic acid) exhibited significantly higher DPPH radical scavenging activities than the DPPH radical scavenging activities of all the studied methanolic plant extracts (*P* < 0.05; Table 3). At all the studied concentrations, the methanolic extract of *A. hockii* produced significantly higher DPPH radical scavenging activities than those recorded for the methanolic extracts of *C. volkensii* and *V. lasiopus* (*P* < 0.05; Table 3). The percentage DPPH radical scavenging activities of the methanolic extracts of *V. lasiopus* and *A. hockii* at a concentration of 0.5 mg/ml were not significantly different (*P* > 0.05); however, the observed percentage radical scavenging activities were significantly higher than that of *C. volkensii* (*P* < 0.05; Table 3).

The concentrations of the studied plant extracts required to scavenge 50% of the DPPH radicals (IC_50_) were also determined in this study. The IC50 values for the *C. volkensii, V. lasiopus,* and *A. hockii* extracts were 0.25 mg/ml, 0.24 mg/ml, and 0.12 mg/ml, respectively. On the other hand, the IC50 value of the standard (L-ascorbic acid) was 0.06 mg/ml.

#### 3.2.3. In Vitro Hydroxyl Radical Scavenging Activities of Methanolic Extracts of *C. volkensii*, *V. lasiopus,* and *A. hockii*

The methanolic extracts of *C. volkensii*, *V. lasiopus,* and *A. hockii* exhibited remarkable in vitro hydroxyl radical scavenging activities (Table 4). At all the tested concentrations, the methanolic extracts of *A. hockii* demonstrated significantly higher hydroxyl radical scavenging activities than those of the methanolic extracts of *C. volkensii* and *V. lasiopus* extracts (*P* < 0.05; Table 4). Moreover, at all tested concentrations, the methanolic extracts of *V. lasiopus* demonstrated significantly higher hydroxyl radical scavenging activities compared to the hydroxyl radical scavenging activities of the methanolic extracts of *C. volkensii*. However, at all the tested concentrations, the in vitro hydroxyl radical scavenging activities of L-ascorbic acid were significantly higher than those of the methanolic extracts of *C. volkensii*, *V. lasiopus,* and *A. hockii* (*P* < 0.05; Table 4).

In this study, the concentration of the studied plant extracts capable of scavenging 50% of the hydroxyl radicals (IC_50_) was also determined. The results showed that the IC50 values for the methanolic extracts of *C. volkensii, V. lasiopus,* and *A. hockii* extracts were 0.11 mg/ml, 0.23 mg/ml, and 0.49 mg/ml, respectively. Besides, the IC50 value of the standard (L-ascorbic acid) was 1.05 mg/ml (Table 4).

### 3.3. Total Phenolic and Flavonoid Contents of Methanolic Extracts of *C. volkensii*, *V. lasiopus,* and *A. hockii*

Determination of the quantity of total phenols of methanolic extracts of *C. volkensii*, *V. lasiopus,* and *A. hockii* was performed in this study. The results showed that the methanolic extract of *A. hockii* had a significantly higher total phenolic content (41.78 ± 0.93 mg of GAE/g) than the phenolic content in the methanolic extracts of *V. lasiopus* (6.04 ± 0.032 mg of GAE/g) and *C. volkensii* (28.51 ± 0.061 mg of GAE/g) (*P* < 0.05; Table 5).

On the other hand, analysis of the total flavonoid content, in methanolic extracts of the studied plant extracts, showed that *A. hockii* contained significantly higher total flavonoids (39.89 ± 0.04 mg of QE/g) compared with the total flavonoid content in the methanolic extracts of *V. lasiopus* (32.89 ± 0.01 mg of QE/g) and *C. volkensii* (22.52 ± 0.09 mg of QE/g) (*P* < 0.05; Table 6).

## 4. Discussion

Increased production of reactive oxygen/nitrogen species and decreased capacity of antioxidant defenses in the body leads to oxidative stress [22, 23]. Generation of reactive oxygen/nitrogen species (ROS/RNS) is inevitable for aerobic organisms and in healthy cells, and it occurs at a controlled rate [24]. Under conditions of oxidative stress, ROS/RNS production is dramatically increased, resulting in subsequent alteration of membrane lipids, proteins, and nucleic acids [6]. Oxidative damage of these biomolecules is associated with aging and a variety of pathological events, including atherosclerosis, carcinogenesis, ischemia reperfusion injury, and neurodegenerative disorders [25].

To maintain homeostasis in the redox system and protect the body against ROS and RNS, humans have evolved complex antioxidant systems, which work to avert deleterious effects of oxidative stress [26]. The body's antioxidant defense systems are of endogenous and exogenous origin [27]. Exogenous sources of antioxidants include *β*-carotene, L-ascorbic acid (vitamin C), *α-*tocopherol, and tocotrienols (vitamin E), which are derived from dietary foods we consume [28]. Endogenous sources of antioxidant defenses include superoxide dismutase (SOD), glutathione peroxidase (GPx), glutathione reductase, and catalase (CAT) enzymes, which catalyze free radical quenching reactions [28–30].

Synthetic antioxidants such as propyl gallate (PG), butylated hydroxyanisole (BHA), and butylated hydroxytoluene (BHT), currently used against oxidative stress, have been associated with adverse health effects including hepatic damages and malignancies. Additionally, they have limited potency in animal models and humans [31]. Currently, there is an upsurge of interest to substitute synthetic antioxidants with naturally occurring antioxidants from plants since they are considerably safer, easily accessible, and affordable [32, 33]. The objective of the current study was to assay for the presence of phytochemicals and the in vitro antioxidant activities of methanolic stem bark extracts of *A. hockii* and leaf extracts of *V. lasiopus* and *C. volkensii*.

Research has indicated that antioxidant activities ought not to be established based on a single antioxidant experimental model [6]. In practice, several in vitro examination techniques are taken into consideration for assessing antioxidant activities [6, 34]. Some of the in vitro antioxidant tests used include hydroxyl scavenging activity, lipid peroxidation inhibition capacity (LPIC), the oxygen radical absorbance capacity (ORAC) method, the nitric oxide scavenging assay, ferric reducing antioxidant power (FRAP), and DPPH scavenging effects [34].

In the present study, the ferric reducing antioxidant power (FRAP) assay was adopted [17]. This method is based on the ability of the analyte to reduce the ferric ion (Fe^3+^) to ferrous ion (Fe^2+^) [35, 36]. Hence, the Fe^2+^ formation can be examined by absorbance capacity at 700 nm [36]. Increases in absorbance at this wavelength indicate an increase in reducing power [36].

The findings of this study demonstrated a concentration-dependent increase in absorbance values of the methanolic extracts of *C. volkensii*, *V. lasiopus,* and *A. hockii* depicting appreciable ferric reducing antioxidant power. The findings were comparable with the in vitro study by Aliyu et al. [15] who demonstrated the antioxidant capacity of the leaves extracts of *Bauhinia rufescens* Lam. Additionally, a study conducted by Adesanoye and Farombi [37] established dose-dependent ferric reducing activity of the methanolic leaf extracts of *Vernonia amygdalina*, in concurrence with our study. Furthermore, our results corroborate with those of Sowndhararajan et al. [38] who demonstrated the antioxidant potential of Indian *Acacia* species methanolic bark extracts.

Moreover, antioxidant activities of the studied plant extracts were appraised according to the criterion of Do et al. [20]. Based on this criterion, all the studied plant extracts were considered as very strong antioxidants since their EC50 values were all below 50 *μ*g/ml. The results that were observed in the study corroborated those of Fidrianny et al. [22] who demonstrated similar effects in *Momordica charantia*.

On the other hand, the DPPH radical scavenging technique described by Brand-Williams et al. [18] has been used across time to evaluate the antioxidant efficacy of various analytes. In this technique, molecules are considered to be antioxidants if they can scavenge for and reduce DPPH free radicals in vitro. The DPPH radical scavenging activity is observed by a characteristic change in colour from blue to yellow, which is quantified at 517 nm [18].

In the current study, we found that DPPH scavenging capacity of the methanolic extracts of *C. volkensii, V. lasiopus,* and *A. hockii* exhibited a concentration-dependent relationship as previously demonstrated by Patil et al. [39] and Vivek et al. [40] in extracts of *Ageratum conyzoides* and *Caesalpinia pulcherrima,* respectively. Moreover, metabolomic profiling of different parts (leaves, flowers, and pods) of *Acacia* species (*Acacia nilotica*, *Acacia seyal,* and *Acacia laeta*) by Abdel-Farid et al. [41] displayed that the antioxidant activities were comparable with those of the studied plant extracts.

Upon grading of the DPPH free radical scavenging effects as per the criterion of Fidrianny et al. [22], all the studied plant extracts were strong antioxidants since their IC_50_ values were lower than 50 mg/ml.

Moreover, the ability of the studied plant extracts to scavenge for the hydroxyl radical was investigated in this study. Research has shown that the hydroxyl radicals directly denature body enzymes via oxidation of thiol (-SH) groups [42]. The hydroxyl radicals are generated through the Fenton reaction: Fe^2+^ + H_2_O_2_ ⟶ Fe^3+^ + OH^−^ + OH^•^ [43]. A sample capable of scavenging for hydroxyl radicals in vitro is considered to be a potent antioxidant with potential effects in vivo.

In this study, the studied plant extracts revealed a concentration-dependent decrease in hydrogen peroxide scavenging activity in agreement with the findings reported on both acetone and aqueous whole plant extracts of *Bulbine abyssinica* [44]. Conversely, some studies have reported a concentration-dependent increase in hydroxyl radical scavenging activities [45]. The concentration-dependent decrease in hydroxyl radical scavenging activity could have been brought about by saturation of reactive centers of hydroxyl radicals by the high extract concentrations leading to low activities, compared to dilute concentrations that ensured easier and rapid reaction, leading to high activity. The half maximal (IC50) values for *A. hockii, C. volkensii,* and *V. lasiopus* were lower than 50 mg/ml, which, according to Fidrianny et al. [22], rendered them very strong.

Extensive research has shown that medicinal plants contain active principles, which are responsible for antioxidant activity [46]. Various phytochemicals of antioxidant value present in medicinal plants are responsible for this bioactivity. Qualitative phytochemical profiling of the studied plant extracts showed the presence of flavonoids, phenols, and tannins among other antioxidant phytochemicals. Quantitative analysis of flavonoids and phenols, the most prominent antioxidant phytochemicals, revealed appreciable amounts, which may have contributed to the antioxidant potency of the studied plant extracts [46].

The antioxidant potential of these phytochemicals is thought to be through the reductive and oxidative capacities that allow absorption and counteracting effects of free radicals [47]. Many of these secondary metabolites are endowed with significant reductive abilities that are attributed to lesser incidences of death and suffering due to oxidative stress-related disorders [48]. Our study highly suggests that phenolics are significant components of these plants, and this is attributed to their biological effects.

Flavonoids are one of the phenolics compounds found in plants, and they are associated with various pharmacological activities including anti-inflammatory and antitumor properties, and they are capable of acting as antioxidants that shield the cells from destructive effects of free radicals [46, 48]. The flavonoids structure, its hydroxyl atom location, and its other properties are important for antioxidant and reactive species neutralizing capacity [49]. These molecules demonstrate potent scavenging effects of destructive radicals that are associated with several disorders [50].

The studied medicinal plants are traditionally used to manage various diseases, which are associated with oxidative stress [8, 11, 12, 14]. Based on the remarkable antioxidant effects demonstrated by the studied plant extracts in the experimented models, the medicinal value of these plants could be exerted through the remediation of oxidative stress. Thus, preliminary investigation of antioxidant activities of methanolic leaf extracts of *C. volkensii* and *V. lasiopus* and stem bark extracts of *A. hockii* we report herein provides a valuable source of biologically active elements and will consequently lead to discovery and development of potent, efficacious, safe, and affordable antioxidants to curb oxidative stress. Furthermore, our study confirms the use *C. volkensii*, *V. lasiopus,* and *A. hockii* in the management of oxidative stress-related disorders in the traditional medicine [11, 12, 14].

## 5. Conclusions and Recommendations

From the obtained results, it was concluded that the methanolic leaf extracts of *Caesalpinia volkensii* and *Vernonia lasiopus* and the methanolic stem bark extract of *Acacia hockii* have appreciable antioxidant capacity and antioxidant-associated phytochemicals. Further studies that aimed at isolating and characterizing the pure phytoactive principles for enhancement are recommended. Toxicity studies on the methanolic stem bark extracts of *Caesalpinia volkensii, Vernonia lasiopus,* and *Acacia hockii* should be performed to determine their safety.

## Figures and Tables

**Table 1:** Qualitative phytochemical composition of methanolic leaf extracts of *C. volkensii* and *V. lasiopus* and stem bark extracts *A. hockii*.

Phytochemicals	*C. volkensii*	*V. lasiopus*	*Acacia hockii*
Flavonoids	+	+	+
Phenols	+	+	+
Steroids	−	−	+
Saponins	+	+	+
Alkaloids	+	+	+
Cardiac glycosides	−	−	+
Terpenoids	+	+	+

+, present; −, absent

**Table 2:** In vitro ferric reducing antioxidant power activities of methanolic extracts of *C. volkensii*, *V. lasiopus*, and *A. hockii*.

Absorbance at 700 nm				
Concentration in (mg/ml)	L-ascorbic acid	*A. hockii*	*V. lasiopus*	*C. volkensii*
**0.2**	1.72 ± 0.01_e_^A^	1.63 ± 0.02_d_^B^	1.35 ± 0.01_e_^C^	1.05 ± 0.02_e_^D^
**0.4**	1.92 ± 0.00_d_^A^	1.82 ± 0.00_c_^B^	1.47 ± 0.01_d_^C^	1.23 ± 0.01_d_^D^
**0.6**	2.25 ± 0.01_c_^A^	1.88 ± 0.01_c_^B^	1.72 ± 0.01_c_^C^	1.42 ± 0.02_c_^D^
**0.8**	2.56 ± 0.02_b_^A^	2.18 ± 0.02_b_^B^	1.87 ± 0.02_b_^C^	1.65 ± 0.01_b_^D^
**1**	2.75 ± 0.00_a_^A^	2.54 ± 0.04_a_^B^	1.93 ± 0.02_a_^C^	1.81 ± 0.01_a_^D^

**EC**_**50**_ **(mg/ml)**	0.14	0.16	0.17	0.21

The values are expressed as mean ± SEM. Values with the same uppercase superscript letter within the same row and those with the same lowercase subscript letter within the same column are not significantly different (*P* > 0.05, one-way ANOVA followed by Tukey's test).

**Table 3:** In vitro DPPH scavenging activities of methanolic extracts of *C. volkensii*, *V. lasiopus*, and *A. hockii.*

DPPH scavenging activity (% inhibition)				
Concentration in mg/ml	Treatments			
	L-ascorbic acid	*C. volkensii*	*V. lasiopus*	*A. hockii*
**0.0625**	46.81 ± 0.46_e_^A^	33.16 ± 0.60_e_^D^	36.44 ± 0.75_e_^C^	39.38 ± 0.30_c_^B^
**0.125**	62.18 ± 0.18_d_^A^	36.10 ± 0.75_d_^D^	44.73 ± 1.73_d_^C^	53.37 ± 1.30_bc_^B^
**0.25**	73.06 ± 1.03_c_^A^	51.64 ± 0.46_c_^C^	50.60 ± 0.17_c_^D^	66.67 ± 1.05_ab_^B^
**0.5**	81.35 ± 0.30_b_^A^	61.31 ± 0.75_b_^C^	70.47 ± 1.79_b_^B^	70.47 ± 1.79_b_^B^
**1**	87.22 ± 0.75_a_^A^	67.36 ± 0.30_a_^D^	76.86 ± 0.96_a_^C^	82.73 ± 0.62_a_^B^

**IC**_**50**_	0.06	0.25	0.24	0.12

The values are expressed as mean ± SEM. Values with the same uppercase superscript letter within the same row and those with the same lowercase subscript letter within the same column are not significantly different (*P* > 0.05, one-way ANOVA followed by Tukey's test).

**Table 4:** In vitro hydroxyl radical scavenging activities of methanolic extracts of C. *volkensii*, *V. lasiopus*, and *A*. *hockii*.

Hydroxyl radical scavenging activity (% inhibition)				
Concentration in mg/ml	L-ascorbic acid	*C. volkensii*	*V. lasiopus*	*A. hockii*
**0.0625**	87.50 ± 1.30_a_^A^	66.99 ± 0.32_a_^D^	73.93 ± 0.91_a_^C^	81.52 ± 0.28_a_^B^
**0.125**	80.56 ± 0.65_b_^A^	56.41 ± 0.37_b_^D^	61.75 ± 0.83_b_^C^	73.56 ± 0.28_b_^B^
**0.25**	73.29 ± 2.03_c_^A^	46.69 ± 0.36_c_^D^	53.74 ± 0.65_c_^C^	63.14 ± 0.56_c_^B^
**0.5**	62.39 ± 0.39_d_^A^	40.71 ± 0.56_d_^D^	47.22 ± 0.21_d_^C^	50.86 ± 0.39_d_^B^
**1**	50.64 ± 0.68_e_^A^	30.98 ± 0.28_e_^D^	38.46 ± 0.56_e_^C^	43.38 ± 0.28_e_^B^

**IC**_**50**_	1.05	0.11	0.23	0.49

The values are expressed as mean ± SEM. Values with the same uppercase superscript letter within the same row and those with the same lowercase subscript letter within the same column are not significantly different (*P* > 0.05, one-way ANOVA followed by Tukey's test).

**Table 5:** Total phenolic contents of methanolic extracts of *C. volkensii*, *V. lasiopus*, and *A. hockii*.

Sample	TPC (mgGAE/g)
*Acacia hockii*	41.78 ± 0.09^a^
*Vernonia lasiopus*	36.04 ± 0.03^b^
*Caesalpinia volkensii*	28.51 ± 0.06^c^

TPC, total phenolic content; mgGAE/g, milligrams gallic acid equivalent per gram of sample. Values are expressed as mean ± SEM. Means with different superscript letters are significantly different by one way ANOVA followed by Tukey's test.

**Table 6:** Total flavonoid contents in the methanolic extracts of *C*. *volkensii*, *V*. *lasiopus*, and *A*. *hockii*.

Sample	TFC (mg QE/g)
*Acacia hockii*	39.89 ± 0.04^a^
*Vernonia lasiopus*	32.89 ± 0.01^b^
*Caesalpinia volkensii*	22.52 ± 0.09^c^

TFC, total flavonoid content; mg QE/g, milligrams of quercetin equivalent per gram of sample. Values are expressed as mean ± SEM. Means with different superscript letters are significantly different by one way ANOVA followed by Tukey's test.

## Data Availability

The data used to support this study are included within this article.
